# Pregnant patient with prune belly syndrome: case report

**DOI:** 10.31744/einstein_journal/2022RC6903

**Published:** 2022-08-15

**Authors:** André Emídio Carvalho Moreno, Mariana Albuquerque Montenegro, Paula Andrade Neiva Santos, Dennyse Araújo Andrade, Laura Alencar Pinto, Manoel Cláudio Azevedo Patrocínio, Júlio Augusto Gurgel Alves

**Affiliations:** 1 Maternidade Escola Assis Chateaubriand Universidade Federal do Ceará Fortaleza CE Brazil Maternidade Escola Assis Chateaubriand, Universidade Federal do Ceará, Fortaleza, CE, Brazil.; 2 Universidade de Fortaleza Fortaleza CE Brazil Universidade de Fortaleza, Fortaleza, CE, Brazil.

**Keywords:** Prune belly syndrome, Pregnancy, high-risk

## Abstract

Prune belly syndrome is a rare congenital disease of unknown etiology that is present in one in every 40 thousand live births, and predominantly affects males, at a ratio of 4:1. In males, it presents with anomalies in the urinary system, absence of abdominal muscles, bilateral cryptorchidism, and infertility. In women, the syndrome has variable presentations, but fertility is preserved. Searching the medical literature, we found only one case of prune belly syndrome in pregnant women. Therefore, the patient in this report is the second case. She was primiparous, 25-years-old, with no abdominal muscles, severe congenital kyphoscoliosis, and pulmonary restriction. Elective cesarean section was performed at 37 weeks of gestation due to maternal risk of uterine rupture by transverse presentation and fetal risk of intrauterine growth restriction. The pre-anesthetic approach defined that general anesthesia might have more risks for the patient due to severe maternal lung disease compared to ultrasound-guided locoregional anesthesia. During prenatal care, there were some maternal complications, such as asthma exacerbations, abdominal pain, and constipation. The newborn was born small for gestational age and this can possibly be explained by maternal restrictive lung capacity. The newborn presented with Apgar score 8/9 and tachypnea, but improved after two hours of life.

## INTRODUCTION

Prune belly syndrome, also called Eagle Barrett syndrome, is a rare congenital disease presenting with anomalies of the urinary system; absence, deficiency, or hypoplasia of abdominal muscles, and bilateral cryptorchidism in men.^(1)^ This congenital malformation has unknown etiology, but, in some cases, there is evidence of a genetic contribution.^(1)^ Prune belly syndrome affects approximately one in 40 thousand live births, with approximately 95% of cases occurring in males and with male infertility.^(2)^ In women, the syndrome has variable presentations, but fertility is preserved.^(2)^ Since the disease is very rare, this is the second case of a pregnant woman with prune belly syndrome in the medical literature.^(2)^

## CASE REPORT

**Figure 1:**
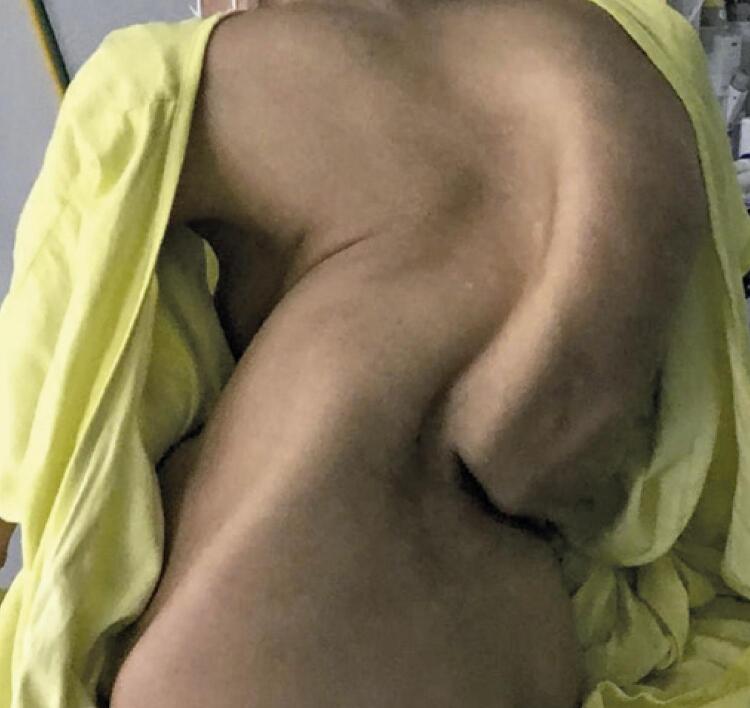
Severe kyphoscoliosis

A 25-year-old primigravida with prune belly syndrome started prenatal care at 12 weeks. At the beginning of pregnancy, the patient weight was 33kg and height of 1.47m. The patient was referred to the high-risk prenatal service due to asthma and severe kyphoscoliosis (Figure 1). She denied consanguinity with her partner, alcoholism, and smoking. Early in pregnancy, she discontinued use of corticosteroids for fear of causing any harm to the baby, and had to be treated once in the emergency room due to an asthma attack. Spirometry showed very severe restrictive lung impairment and no response to bronchodilator. After it was clarified that the asthma treatment would not harm the fetus, the patient started to use the medication correctly, without any further worsening of the condition. The patient had no urinary tract malformations and no urinary tract infection during gestation.

**Figure 2:**
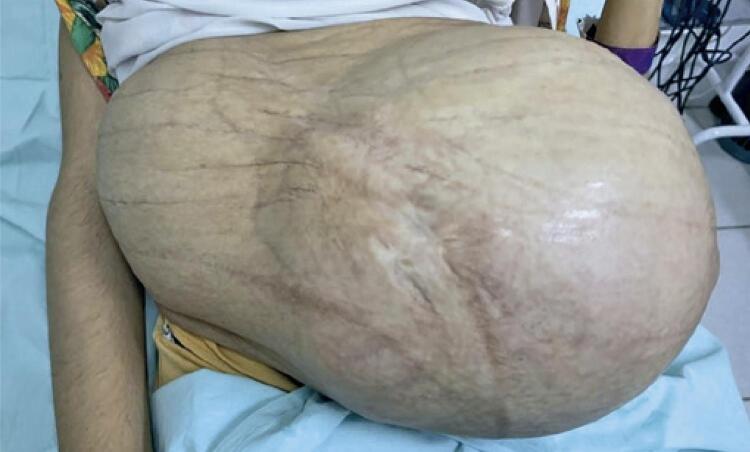
Presentation of the patient’s pregnant abdomen

**Figure 3:**
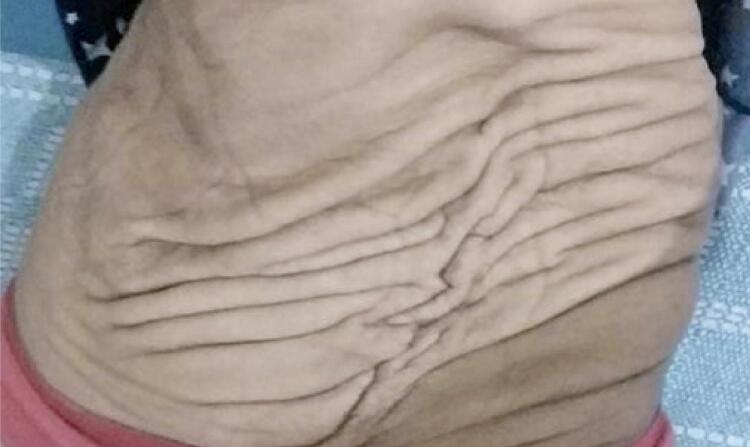
Presentation of the abdomen with hypoplasia of the abdominal muscles in puerperium

With the progressive development of the uterus (Figure 2) associated with hypoplasia of the abdominal muscles (Figure 3), the abdominal organs were compressed and displaced, which resulted in frequent constipation, abdominal bloating, and epigastric pain. The patient gained only 3.4kg during gestation due to abdominal discomfort and gastroesophageal reflux.

Elective cesarean section was scheduled for 37 weeks due to the possibility of intrauterine growth restriction, with fetal weight approximately below the 3^rd^ percentile, observed in one of the prenatal ultrasound examinations. In this case, a pre-anesthetic consultation was performed, in which ultrasound-guided locoregional anesthesia was preferred, to avoid the higher risks of general anesthesia in a patient with lung disease. Corticotherapy had been prescribed since 34 weeks because of the imminent risk of a premature delivery.

**Figure 4:**
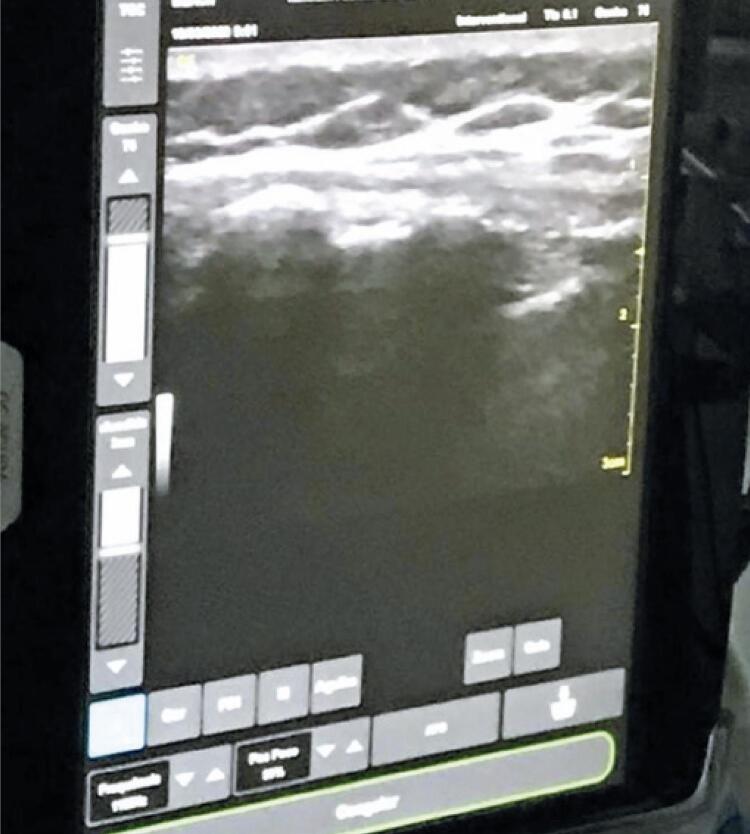
Ultrasound with linear transducer for locoregional anesthesia

The cesarean section was performed with the patient in dorsal decubitus under spinal anesthesia guided by point-of-care ultrasound (POCUS) (Figure 4). A Pfannenstiel incision was made and the abdominal wall was dissected by layers, noting the absence of abdominal muscles and typical aponeurosis leaflets. In addition, segmental arciform hysterectomy was performed and a live male conceptus was removed; abundant, clear amniotic fluid was observed. The appendages were macroscopically normal. During the surgery, a copper intrauterine device (IUD) was inserted with the patient’s prior consent.

The newborn did not require resuscitation, and nasopharyngeal and endotracheal aspiration was performed. With 2,370kg, length of 46cm, 5^th^ percentile, and Apgar score 8/9, the newborn presented with respiratory distress due to probable transient tachypnea. He presented no malformations on physical examination. Continuous positive airway pressure (CPAP) was indicated, with improvement after 2 hours. Therefore, the newborn evolved satisfactorily well.

This study was approved by the Research Ethics Committee of *Maternidade Escola Assis Chateaubriand* of the *Universidade Federal do Ceará* under # 4.492.377, CAAE: 41301220.2.0000.5050.

## DISCUSSION

Prune belly syndrome is a rare condition with sporadic incidence in most cases, although familial cases have been described. The pathophysiology is possibly characterized by an alteration in the intermediate-lateral plate of the mesoderm or genital prominence. The absence of fetal abdominal musculature caused spinal instability and chest deformity in the patient of this case, compromising lung development.^(1,3)^

Also, a maternal pelvic rotation did not allow for vaginal insinuation or descent of the fetus, which, together with the transverse presentation, was responsible for the indication of a cesarean section. Elective preterm delivery occurred due to the maternal risks of uterine rupture and the risks of fetal distress due to fetal weight near the 3^rd^ percentile. A major difficulty would be the choice of the type of anesthesia, due to the anatomical deformity of the spine that made it difficult to perform locoregional anesthesia. On the other hand, general anesthesia would cause serious risks to the patient due to restrictive pulmonary insufficiency and asthma. Therefore, we opted for locoregional anesthesia with ultrasound-guided puncture.

The newborn’s weight was low for gestational age. Protective factors, such as good schooling; absence of smoking, alcoholism, and use of illicit drugs; adequate prenatal care; absence of infections; vascular diseases, and hypertensive diseases of pregnancy, were not sufficient to prevent the inappropriate development of the fetus. Possibly, the low maternal weight when she got pregnant (body mass index of 15.27), the inadequate maternal weight gain during pregnancy, the severe maternal kyphoscoliosis associated with restricted lung functional capacity were the factors that may have contributed to chronic fetal hypoxia.^(4,5)^

## CONCLUSION

Prune belly syndrome is a rare disease that, despite the congenital malformations presented by the patient, allowed a good evolution of the pregnancy, in addition to a newborn with good vitality and absence of complications during the puerperium. A premature abdominal delivery was chosen due to the maternal and fetal risks.
